# Patterns of work-related stress and their predictors among emergency department nurses and emergency medical services staff in a time of crisis: a latent profile analysis

**DOI:** 10.1186/s12912-023-01241-9

**Published:** 2023-04-06

**Authors:** Edyta Charzyńska, Aghil Habibi Soola, Naser Mozaffari, Alireza Mirzaei

**Affiliations:** 1 Faculty of Social Sciences, University of Silesia in Katowice, Katowice, Poland; 2 Department of Nursing, School of Nursing and Midwifery, Ardabil University of Medical Sciences, Ardabil, Iran; 3 Department of Emergency Nursing, School of Nursing and Midwifery, Ardabil University of Medical Sciences, Ardabil, Iran

**Keywords:** Work-related stress, Healthcare workers, Emergency department nurses, Emergency medical services staff, COVID-19

## Abstract

**Background:**

Previous studies have shown that a disease outbreak may cause high stress among healthcare workers. However, the vast majority of those studies applied a variable-centered approach, in which relationships between the variables are believed to be identical across the studied population. The main purpose of this study was to identify latent profiles of healthcare workers with similar combinations of levels of various work-related stressors during the coronavirus disease 2019 (COVID-19) pandemic and to examine their predictors.

**Methods:**

A cross-sectional paper-and-pencil study was conducted among a convenience sample of 297 emergency department (ED) nurses and 219 emergency medical services (EMS) staff members working in 10 hospital EDs and 52 EMS centers in Ardabil province, Iran. Data were collected using the Health and Safety Executive Management Standards Indicator Tool (HSE-MS IT).

**Results:**

Using the latent profile analysis (LPA), five work-related stress profiles were identified: “high stress with a good understanding of one’s job role” (11.1%), “moderate stress” (41.9%), “relatively high stress with average demands and a very low understanding of one’s job role” (23.8%), “low stress” (18.0%), and “generally low stress but with very high job demands and relational conflicts” (5.2%). Age, marital status, service location, workplace, and the number of overtime hours significantly predicted profile membership.

**Conclusion:**

The results of the study suggest the importance of incorporating various sources of stress and using the person-centered approach when investigating the work-related stress of healthcare workers during disease outbreaks. Identifying sociodemographic and work-related predictors of profile membership may be useful for preparing interventions that will be better suited to healthcare workers’ needs.

## Introduction

Work-related stress—also known as occupational stress—is an alarming phenomenon worldwide and has been acknowledged as a major public health problem due to its negative impact on the physical, mental, emotional, and psychological well-being of employees in various occupations [1]. The National Institute for Occupational Safety and Health [2] defines work-related stress as a harmful physical and mental response resulting from an incompatibility between job requirements and the abilities, support resources, and needs of the employee. In a similar vein, the Job-Demand Resources (JD-R) model [3] states that stress may occur if an individual feels that they cannot meet the demands placed on them within the context of their job role.

Although work-related stress is present in all professions, healthcare workers are particularly susceptible to experiencing a lot of stress and pressure in their work environment [4, 5]. Critical situations such as the outbreak of the coronavirus disease 2019 (COVID-19) add even more demands and burdens to the already demanding profession, causing a threat to the mental health and well-being of healthcare workers [6, 7].

The first case of COVID-19 was reported in Wuhan, China, in December 2019, and then quickly spread in almost all countries. Iran reported the first confirmed case of COVID-19 in Qom on February 19, 2020, and became the second focal point for the spread of COVID-19 in the world [8]. At the time of data collection in the current study (the beginning of January 2021), over 1,280,000 cases have been reported in Iran, with the death toll of above 56,000, making it one of the hardest-hit countries as regards the COVID-19 pandemic [9].

The outbreak of the COVID-19 pandemic severely strained healthcare systems worldwide in unprecedented ways [10]. Healthcare workers had to face various sources of stress, such as shortages of materials and protective equipment, high demand for hospital services, reduced availability of trained personnel, increased number of deaths, fear of infection, and fear of transmitting the infection to others [8, 11, 12]. As a consequence of high demands, insufficient resources, uncertainty, and the resultant stress, healthcare workers reported decreased levels of quality of life [8, 13] and increased levels of anxiety, depression, fatigue, insomnia, burnout, and turnover intention [6, 12, 14].

Considering the need for adequate psychological support for healthcare professionals, especially in a time of crisis, in the current study we focus on work-related stress among emergency department (ED) nurses and emergency medical services (EMS) staff, who have been at the forefront of patient care during the pandemic. Identifying the patterns of work-related stress among ED nurses and EMS staff may help promote their well-being and ensure the standards of healthcare services and the quality of patient care.

## Measuring multiple work-related stressors

Work-related stress may be triggered by various stressors, including organizational factors, such as high or low demands, lack of autonomy at work, or tense relationships with coworkers. Given the fact that sources of stress are frequently interrelated, it is necessary to investigate different work-related stressors and examine their relationships with each other. Meeting this need, the UK Health and Safety Executive (HSE) has developed the Management Standards (MS) to anticipate and prevent long-term negative consequences of work-related stress for individuals and organizations [15, 16]. The MS encompass the key six areas widely recognized as potential causes of work-related stress: (1) *Demands*: workload, company requirements, and task assignments; (2) *Control*: the perceived control of organizing and managing one’s own job activities; (3) *Support*: the level of encouragement, sponsorship, and resources provided by the organization, line management, and work colleagues; (4) *Relationships*: promoting good atmosphere at work to prevent interpersonal conflicts and deal with unacceptable behaviors; (5) *Role*: whether people understand their role and responsibilities within the organization and whether the organization ensures that the employees do not have conflicting roles; and (6) *Change*: how organizational change is managed and communicated within the organization. Based on this standard-oriented model, the same agency [15, 16] developed the Health and Safety Executive Management Standards Indicator Tool (HSE-MS IT), which has been widely used in research on work-related stress [16–18].

A common drawback of most previous studies on work-related stress among healthcare workers that used the HSE-MS IT is that they apply the variable-centered approach, which is based on the assumption of population homogeneity and focuses on explaining relationships between variables of interest in a population. By contrast, in the person-centered approach, the focus lies on the identification of latent subpopulations of individuals based on several observed characteristics (i.e., indicators), which gives this approach a higher level of specificity compared to the variable-centered one [19]. Instead of emphasizing individual variables, this view allows the researcher to identify different constellations of constructs and thus gain more knowledge about the relationships between various work-related characteristics. For example, although experiencing high support at work is often related to the feeling that the organization takes care of promoting positive relationships between coworkers and excluding unacceptable behaviors, it does not have to be the case; some employees may feel adequately supported in terms of work-related duties and responsibilities but at the same time experience interpersonal tensions or aggressive behaviors from their coworkers. The variable-centered approach shows just the general, “average” picture, putting aside individual experiences [20]. Using the person-centered approach in the current study may help fill this gap by providing a more comprehensive picture of the relationships between work-related stressors. Moreover, by identifying the predictors of latent profiles of work-related stress, this approach may provide knowledge that will help prepare tailored interventions aimed at reducing work-related stress among healthcare workers.

## Aim of the study

Most studies using the HSE-MS IT in healthcare settings have explored each potential source of stress separately, in this way missing potentially meaningful information on the patterns of work-related stress in distinct subgroups of healthcare workers [18, 21, 22]. One of the exceptions is the study by Portoghese et al. [23], carried out among 1,671 healthcare workers in Italy, in which the person-centered approach was used to identify patterns of job types. However, due to the study aim, only four subscales of the HSE-MS IT were included: Demands, Control, Managerial Support, and Coworkers’ Support [23]. Importantly, there is some evidence that including the remaining HSE-MS IT subscales (i.e., Change, Role, and Relationships) may be fruitful and provide additional information about healthcare workers’ characteristics and functioning [24, 25]. This seems to be especially true in the midst of a pandemic, when many organizational changes forced by unstable and unsafe circumstances are often related to the confusion of job roles, unpreparedness for changes, and interpersonal conflicts at the workplace [26].

Based on the above theoretical and empirical premises, the main purpose of the current study was to identify latent profiles of ED nurses and EMS staff with similar combinations of levels of work-related stressors during the COVID-19 pandemic, using all the subscales of the HSE-MS IT. In other words, the study aimed to find out whether the sample of ED nurses and EMS staff was heterogeneous in terms of work-related stressors. Moreover, to expand the current knowledge on the predictors of work-related stress, we examined the relationships between the latent profile membership and sociodemographic and work-related variables.

## Research hypotheses

We expected that different subtypes of work-related stressors existed among healthcare workers during the COVID-19 pandemic. Specifically, we hypothesized that we would identify profiles with homogenous levels of all work-related stressors (i.e., with low/moderate/high levels of stress indicators) and one or more profiles with more diversified levels of work-related stressors. In addition, we expected that sociodemographic (i.e., sex, age, and marital status) and work-related (i.e., service location, workplace, and the number of overtime hours per month) variables would predict profile membership.

## Methods

### Sample and setting

A cross-sectional paper-and-pencil study was conducted to recruit participants. The sample was collected between January and March 2021 using convenience sampling among ED nurses and EMS staff of the Ardabil University of Medical Sciences in the Ardabil province, Iran.

According to the information received from the Office of Treatment Supervision, the Ardabil University of Medical Sciences has 10 hospital EDs and 52 EMS centers. Before approaching these centers, a letter of recommendation was obtained from the university’s vice chancellor for research. The letter was then presented to the officials of EDs and EMS centers. Next, the nursing officers of those institutions introduced the research team members to the ED nurses and EMS staff.

The inclusion criteria for the study were as follows: (1) being an ED nurse or a member of EMS staff, (2) having at least six months of working experience in EDs or EMS centers, (3) not being on leave for at least a week before completing the questionnaire, and (4) having no history of mental disorders. Based on the response to the inquiry sent to the nursing offices and the EMS centers of the Ardabil University of Medical Sciences, out of 926 ED nurses and EMS staff working in all 10 hospital EDs and 52 EMS centers of the Ardabil University of Medical Sciences, 105 persons were not eligible to participate in the study because of failing to meet the inclusion criteria no. (2) and/or (3). Therefore, a questionnaire was distributed among 821 participants (467 ED nurses and 354 members of EMS staff) by the research team members. No incentives were provided to the participants.

Out of 821 questionnaires distributed among the participants, 550 were returned. Data from 34 persons were excluded from the analysis: 28 were removed due to the high rate of missing responses (at least 50% missing values), and 6 due to failure to meet inclusion criterion no. (4). Therefore, the final sample consisted of 516 participants (63% response rate), including 297 ED nurses and 219 EMS staff members.

### Instruments

The level of work-related stress was measured with the Persian version [27] of the HSE-MS IT [15, 16]. It includes 35 items that make up 7 subscales related to primary stressors at the workplace: (1) Demands (eight items, e.g., “I have to work very fast.”); (2) Control (six items, e.g., “I have a choice in deciding how I do my work.”); (3) Manager’s support (five items, e.g., “I can rely on my line manager to help me out with a work problem.”); (4) Peer support (four items, e.g., “I get help and support I need from colleagues.”); (5) Relationships (four items, e.g., “There is friction or anger between colleagues.”); (6) Role (five items, e.g., “I am clear what my duties and responsibilities are.”), and (7) Change (three items, e.g., “When changes are made at work, I am clear how they will work out in practice.”). The subscales “Manager’s support” and “Work colleague support” cover the same HSE MS (called: “Support”) and are closely related to each other, and thus were combined before conducting further analyses. The responses to all the items are given on a five-point Likert scale (1 = “never,” 2 = “rarely,” 3 = “sometimes,” 4 = “often,” and 5 = “always”). Twelve items need to be reverse-scored. High scores represent low levels of pressure and stress, and low scores represent high levels of pressure and stress. In the current study, the reliability coefficients calculated using Cronbach’s alpha ranged from 0.64 (Control) to 0.82 (Support).

### Ethical considerations

The research obtained ethical approval from the Ethics Committee of the Ardabil University of Medical Sciences (IR.ARUMS.REC.1399.459). Informed consent was obtained from all participants before they started filling out the questionnaire. Participants were informed that their participation was voluntary, that their responses were anonymous, and that they could withdraw from the study at any time without giving any reason.

### Data analysis

In the first step of the analysis, descriptive statistics, reliability scores, and bivariate correlations between the indicators were calculated. We also compared the levels of work-related stress between ED nurses and EMS staff using a *t*-test for two independent samples.

To identify latent profiles with distinct patterns of work-related stress and their predictors, we used the latent profile analysis (LPA), which belongs to the family of person-centered approaches and is suitable for continuous variables. To avoid affecting the composition of latent profiles by covariates, we employed a three-step approach [28]. In the first step of the LPA, between one and six models were built and tested using the maximum likelihood with robust standard errors (MLR) estimator. All models used 3,000 random sets of start values, 100 iterations for each random start, and the 200 best solutions retained for final stage optimization. To identify the optimal number of profiles, we used the Bayesian Information Criterion (BIC), the Akaike Information Criterion (AIC), and the sample-size adjusted BIC (SABIC) [29]. Lower values of these indices suggest a better model fit. Moreover, the Vuong-Lo-Mendell-Rubin test (VLMR), Lo-Mendell-Rubin adjusted LRT test (LMR-LRT), and bootstrap likelihood ratio test (BLRT) were used to compare a (*k*-1)-cluster model (the null model) with a *k*-cluster model (the alternative, less restrictive model). A statistically significant *p*-value for these tests suggests the *k*-cluster model fits the data better than a model with one fewer cluster [30].

When selecting the optimal model, we also considered model parsimony (favoring less complex models) and the size of the profiles (at least 5% of the total sample to exclude artificial and non-replicable profiles). Moreover, we took into account the substantive interpretability of each solution to check if the profiles truly represent distinct categories that differ from each other qualitatively, not merely quantitatively [31]. In this way, we made sure that adding another profile would bring some new information to the model. In addition to these criteria, we calculated the entropy value and average posterior probability for each class to assess the accuracy with which the participants were classified into latent profiles. The value of entropy ≥ 0.8 indicates the “good” separation of the profiles; average posterior probability > 0.7 suggests that the classification accuracy is adequate [32]. All analyses were performed using the Mplus version 8 [33].

After identifying the optimal solution, the participants were assigned to latent profiles based on the probability scores (step 2). In the last step, these probability scores were related to sociodemographic (sex, age, and marital status) and work-related (service location, workplace, number of overtime hours per month) characteristics, which were examined as predictors of latent profile membership. For this purpose, we used the three-step method implemented by Mplus through the “auxiliary” (R3STEP) command.

## Results

### Participants’ characteristics

Table 1 presents the sociodemographic characteristics of the participants. The sample consisted of 297 (57.6%) ED nurses and 219 (42.4%) EMS staff members, including 129 EMS nurses, 72 EM technicians, and 18 EM dispatchers. Most participants were men (60.3%), were in the age range of 21–30 (51.0%), had a bachelor’s degree (83.9%), were married (58.9%), and worked in Ardabil city (54.7%). Moreover, about 3 out of 5 participants (61.6%) had worked between 41 and 120 overtime hours in the preceding month.

**Table 1:** Sociodemographic and work-related characteristics of the healthcare workers (N = 516)

Characteristic	Categories	n (%)
Sex	Male Female	311 (60.3%) 205 (39.7%)
Age (years)	21–30 31–40 > 40	263 (51.0%) 195 (37.8%) 58 (11.2%)
Education	Associate’s degree Bachelor’s degree	83 (16.1%) 433 (83.9%)
Marital status	Unmarried Married	212 (41.1%) 304 (58.9%)
Workplace	Emergency department Emergency medical service	297 (57.6%) 219 (42.4%)
Service location	Ardabil city Countryside	282 (54.7%) 234 (45.3%)
Overtime hours in the last month	≤ 40 41–80 81–120 > 120	117 (22.7%) 171 (33.1%) 147 (28.5%) 81 (15.7%)

Note. *M* = mean; *SD* = standard deviation.

### Descriptive statistics and correlations

Table 2 presents the descriptive statistics, reliability, and correlations between the work-related stressors. Most work-related stressors were positively related to each other, except for the relationships between demands and control and role, which were insignificant.

**Table 2:** Descriptive statistics, reliability, and correlations between the subscales of the HSE-MS IT (N = 516)

Subscales	M	SD	α	1.	2.	3.	4.	5.	6.	7.
1. Demands	2.96	0.65	0.71	1						
2. Control	3.36	0.69	0.64	−0.02	1					
3. Support	3.34	0.74	0.82	0.10*	0.58***	1				
4. Relationships	3.19	0.85	0.64	0.55***	0.15***	0.28***	1			
5. Role	3.89	0.81	0.76	−0.03	0.52***	0.48***	0.15***	1		
6. Change	3.23	0.89	0.65	0.12**	0.55***	0.67***	0.21***	0.37***	1	
7. Stress (total)	3.33	0.52	0.87	0.37**	0.70***	0.86***	0.55***	0.64***	0.76***	1

Note. *M* = mean; *SD* = standard deviation; α = Cronbach’s alpha. Higher means indicate lower work-related stress. *p < .05, **p < .01, ***p < .001

### Comparison of work-related stress between ED nurses and EMS staff

Table 3 shows the results of the comparison of ED nurses and EMS staff in terms of work-related stress. The former group had higher levels of stress for all indicators except demands than the latter group (Table 3).

### Latent profile analysis

#### Latent profiles

The comparison of six LPA models for work-related stress is shown in Table 4. BIC, AIC, and SABIC values decreased with the addition of latent profiles. The comparison of model fit criteria suggested that the five-profile solution fit the data best. Although the results of BLRT were not very informative, the results of the VLMR and LMR-LRT supported the five-profile solution over the four-profile solution. Moreover, the inspection of four-profile and five-profile solutions showed that adding the fifth profile provided new information and allowed for identifying meaningful and interpretable profiles. Considering all the above, we ultimately chose the five-profile solution as best fitting the data. Five profiles were well separated, which was indicated by a high entropy value (0.81) and the average posterior probabilities higher than 0.7 for each profile (Profile 1: 0.89; Profile 2: 86; Profile 3: 0.90, Profile 4: 0.91, and Profile 5: 0.90).

**Table 3:** Comparison of the levels of work-related stress between ED nurses (n = 297) and EMS staff (n = 219)

Subscales	ED nurses		EMS staff		t-test
	M	SD	M	SD	
Demands	2.94	0.65	2.99	0.67	−0.86
Control	3.23	0.67	3.55	0.69	−5.42***
Support	3.27	0.70	3.46	0.80	−2.86**
Relationships	3.13	0.81	3.29	0.91	−2.04*
Role	3.73	0.86	4.13	0.69	−5.82***
Change	3.15	0.84	3.35	0.96	−2.49*
Stress (total)	3.25	0.47	3.46	0.58	−4.57***

Note. *M* = mean; *SD* = standard deviation. Higher means indicate lower work-related stress. *p < .05, **p < .01, ***p < .001

**Fig. 1:**
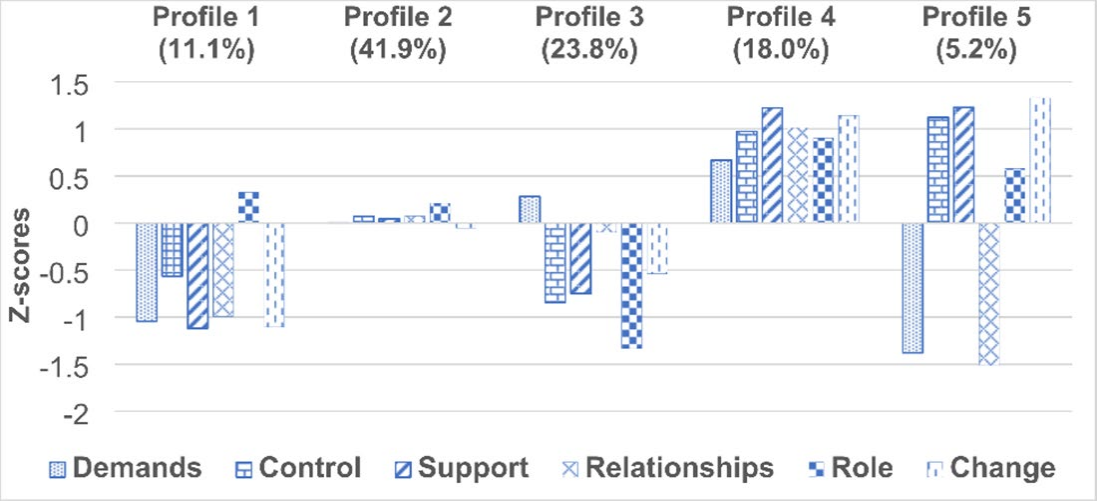
Five-profile model of work-related stress (N *=* 516). Note. *Z*-scores: standard scores. Positive *z*-scores indicate lower-than-average work-related stress in a given domain, whereas negative *z*-scores indicate higher-than-average work-related stress in a given domain

Figure 1 presents five latent profiles identified by the LPA. Profile 1 (11.1%) grouped persons with high levels of stress in all HSE-MS IT subscales except Role (“high stress with a good understanding of one’s job role”). The most numerous Profile 2 (41.9%) consisted of employees with average levels of stress for all indicators (“moderate stress”). Members of Profile 3 (23.8%) had an average level of job demands combined with a very low level of role orientation and low levels of job control, perceived support, and the management of organizational change (“relatively high stress with average job demands and a very low understanding of one’s job role”). Profile 4 (18.0%) was characterized by very low levels of stress for all indicators (“low stress”). The least numerous (5.2%) Profile 5 consisted of persons who felt high control and high support in their job tasks, understood their role in the organization, and felt that the organizational change was well managed, but declared high job demands and conflicted relationships with coworkers (“generally low stress but with very high work demands and relational conflicts”).

**Table 4:** Fit indices for the latent profile analysis of work-related stress (N = 516)

Model	BIC	AIC	SABIC	Entropy	Smallest profile size		VLMR (*p-*value)	LMR-LRT (*p-*value)	BLRT (*p-*value)
1-profile	8855.02	8804.06	8816.93	–	–		–	–	–
2-profile	8303.00	8222.32	8242.69	0.814	0.371		< 0.001	< 0.001	< 0.001
3-profile	8210.84	8100.44	8128.31	0.761	0.222		0.069	0.072	< 0.001
4-profile	8120.51	7980.39	8015.76	0.797	0.054		0.196	0.202	< 0.001
**5-profile**	**8009.12**	**7839.28**	**7882.16**	**0.814**	**0.052**		**0.007**	**0.008**	**< 0.001**
6-profile	Not well-identified								

Note. BIC: Bayesian Information Criterion; AIC: Akaike Information Criterion; SABIC: sample-size adjusted BIC; VLMR = Vuong-Lo-Mendell-Rubin test; LMR-LRT = Lo-Mendell-Rubin adjusted LRT test; BLRT = bootstrap likelihood ratio test. Bold values represent a best-fitting model

#### Predictors of latent profile membership

Table 5 presents the results of testing sociodemographic and work-related variables as predictors of latent profile membership. There were no differences among profiles in terms of sex. As for age, members of Profile 4 (“low stress”) were younger than members of Profile 1 (“high stress with a good understanding of one’s job role”) and Profile 5 (“generally low stress but with very high job demands and relational conflicts”). Moreover, members of Profile 2 (“moderate stress”) were younger than members of Profile 1 (“high stress with a good understanding of one’s job role”). The proportion of unmarried to married persons was higher in Profile 5 (“generally low stress but with very high job demands and relational conflicts”) than in Profile 1 (“high stress with a good understanding of one’s job role”) and Profile 3 (“relatively high stress with average job demands and a very low understanding of one’s job role”). The proportion of persons working in Ardabil city to persons working in the countryside was higher in Profile 2 (“moderate stress”) and Profile 3 (“relatively high stress with average job demands and a very low understanding of one’s job role”) than in Profile 1 (“high stress with a good understanding of one’s job role”). Moreover, the proportion of EMS staff to ED nurses was lower in Profile 3 (“relatively high stress with average job demands and a very low understanding of one’s job role”) than in Profile 1 (“high stress with a good understanding of one’s job role”), Profile 2 (“moderate stress”), and Profile 4 (“low stress”). In addition, members of Profile 3 (“relatively high stress with average job demands and a very low understanding of one’s job role”) had more hours of overtime per month compared to members of Profile 2 (“moderate stress”), Profile 4 (“low stress”), and Profile 5 (“generally low stress but with very high job demands and relational conflicts”).

**Table 5:** Relationships between the latent profile membership and sociodemographic and work-related variables (N = 516)

Predictors	Comparison of latent profiles									
	Ref. Profile: 1				Ref. Profile: 2			Ref. Profile: 3		Ref. Profile: 4
	Profile 2	Profile 3	Profile 4	Profile 5	Profile 3	Profile 4	Profile 5	Profile 4	Profile 5	Profile 5
Sex	−0.14 (0.54)	−0.39 (0.51)	−0.89 (0.57)	−0.36 (0.69)	−0.25 (0.37)	−0.74 (0.47)	−0.22 (0.59)	−0.50 (0.43)	0.03 (0.58)	0.53 (0.65)
Age	−0.65 (0.30)*	−0.60 (0.32)	−1.22 (0.37)**	−0.36 (0.38)	0.05 (0.25)	−0.58 (0.32)	0.29 (0.33)	−0.63 (0.32)	0.23 (0.34)	0.86 (0.41)*
Marital status	−0.38 (0.47)	−0.22 (0.52)	−0.50 (0.49)	−1.23 (0.61)*	0.17 (0.37)	−0.11 (0.33)	−0.85 (0.48)	−0.28 (0.39)	−1.01 (0.50)*	−0.73 (0.52)
Service location	−0.98 (0.45)*	−1.34 (0.47)**	−0.87 (0.47)	−1.08 (0.61)	−0.36 (0.34)	0.10 (0.31)	−0.11 (0.52)	−0.47 (0.36)	0.26 (0.54)	−0.21 (0.55)
Workplace	0.26 (0.57)	−1.96 (0.65)**	0.08 (0.58)	−0.71 (0.79)	−2.22 (0.51)***	−0.18 (0.41)	−0.97 (0.69)	2.04 (0.52)***	1.25 (0.76)	−0.79 (0.71)
Overtime hours	−0.24 (0.20)	0.16 (0.22)	−0.48 (0.23)	−0.57 (0.36)	0.41 (0.18)*	−0.23 (0.18)	−0.33 (0.33)	−0.64 (0.21)**	−0.73 (0.34)*	−0.09 (0.35)

Note. Values are estimates from the R3STEP multinomial logistic regression analysis with standard errors in parentheses. *Ref =* reference profile. *p < .05, **p < .01, ***p < .001. Positive values indicate greater likelihood of membership in the given profile compared to the reference profile, and negative values indicate greater likelihood of membership in the reference profile compared to the given profile. All significant coefficients are bolded. Sex (0 = male, 1 = female), marital status (0 = unmarried, 1 = married), service location (Ardabil = 0, countryside = 1), workplace (0 = ED nurses, 1 = EMS staff). Profile 1: “high stress with a good understanding of one’s job role,” Profile 2: “moderate stress,” Profile 3: “relatively high stress with average job demands and a very low understanding of one’s job role,” Profile 4: “low stress,” Profile 5: “generally low stress but with very high job demands and relational conflicts.”

## Discussion

### Latent profiles of work-related stress and their predictors

Given the huge strain and heavy workload of healthcare workers, especially during disease outbreaks [6, 12, 34], it is of utmost importance to comprehensively investigate their work-related stress levels. The purpose of the current study was to establish whether distinct profiles of healthcare workers could be identified on the basis of their work-related stress levels during the COVID-19 pandemic, and if so, to examine sociodemographic and work-related predictors of profile membership.

Five latent profiles were identified based on the model fit indices and interpretability. Two of these profiles were homogenous: Profile 4 with low levels of stress (18.0%), and the most numerous Profile 2 (41.9%) with moderate levels of stress. The members of Profile 4 were younger than members of Profile 1 (“high stress with a good understanding of one’s job role”) and Profile 5 (“generally low stress but with very high job demands and relational conflicts”). The evidence on the role of age in work-related stress in healthcare workers is inconclusive [1, 35]. In demanding situations such as the pandemic, increased stress may occur in older healthcare workers due to age-related burnout and decreased physical capacity to work intensively [35]. Moreover, with increasing age, healthcare workers’ ability to adapt and tolerate a lack or insufficiency of job resources (such as autonomy on the job or feeling respected and fairly treated) may decrease, leading to higher work-related stress [36].

The proportion of ED nurses to EMS staff was lower in profiles with moderate and lower levels of stress (i.e., Profiles 2 and 4) compared to Profile 3 (“relatively high stress with average demands and a very low understanding of one’s job role”). This result is supported by ED nurses’ higher levels of work-related stress for all domains except demands compared to EMS staff (see Table 3). These findings suggest that despite similar job demands, ED nurses may be more susceptible to increased stress during the pandemic than EMS staff due to lower job resources, such as poorer managers’ and colleagues’ support, lower job control, and less clear roles and responsibilities (see also [11]).

In addition, members of Profiles 2 and 4 had fewer overtime hours per month than those in Profile 3 (“relatively high stress with average demands and a very low understanding of one’s job role”). This result is consistent with studies based on the variable-centered approach, which showed a positive relationship between the number of overtime hours and stress level [37, 38]. The tremendous growth of the number of patients requiring care and treatment during the COVID-19 pandemic has significantly increased the workload of healthcare workers. What is more, some nurses had to go on sick leave due to the COVID-19 infection, which additionally contributed to the shortage of nursing staff. In such conditions, the remaining nurses had to work longer hours, which may have increased their work-related stress [1, 39].

The remaining three profiles (i.e., Profiles 1, 3, and 5) turned out to be more diversified in terms of work-related stress than Profiles 2 and 4. The common feature of members of Profiles 1 and 3 is a relatively high total level of stress (see Fig. 1). The main difference between these profiles lies in the levels of demands and role clarity: despite high demands, members of Profile 1 understood their role and responsibilities quite well, knew their duties and tasks, and generally did not experience conflicting roles. By contrast, members of Profile 3 did not feel overworked but perceived their role as very unclear and ill-defined, with job tasks often extending their regular duties. Interestingly, in this profile, there was a higher proportion of ED nurses to EMS staff and a higher proportion of persons working in Ardabil city to those working in the countryside compared to Profile 1. During the pandemic, ED nurses and persons working in a large city may often face problems with role transparency, which may be related particularly to the lack of specific protocols and procedures in critical situations and staff shortages, resulting in the assignment of new tasks and duties, often requiring greater experience, skills and abilities than one possesses [40]. In a crisis, ED nurses may also experience a role conflict stemming from the multiple and contradictory demands from supervisors, managers, doctors, and the executive staff [22, 41]. The importance of role clarity in the stress level of healthcare workers was supported in a study conducted during the severe acute respiratory syndrome (SARS) epidemic in Hong Kong by Lam et al. [25]. In that study, emergency room nurses reported that despite sufficient knowledge of their respective tasks, focusing on monitoring and preventing the disease instead of saving lives was related to conflict and role ambiguity, which in turn led them to stress. The results of the current study suggest that despite many challenges and unexpected and dangerous events often faced by EMS staff, their tasks and duties during the pandemic may be better structured and less conflicting than those performed by ED nurses. The importance of role clarity needs further investigation, especially since some data suggest that the role conflict may be the most important cause of job dissatisfaction among nurses [42].

The least numerous Profile 5 was characterized by low levels of stress in most domains, except for demands and relationships, for which it was high. Interestingly, this profile included a higher proportion of single persons to married persons than Profile 1 (“high stress with a good understanding of one’s job role”) and Profile 3 (“relatively high stress with average demands and a very low understanding of one’s job role”). This result sheds some light on the contradictory findings of studies using the variable-centered approach concerning the role of marital status in work-related stress. On the one hand, in the present study, the proportion of married persons to single persons was higher for profiles with relatively high levels of stress (i.e., Profiles 1 and 3) than in Profile 5 (see Fig. 1). This result corroborates studies showing a positive correlation between being married and having a greater level of work-related stress, which may be primarily related to work-family conflicts [43]. On the other hand, single persons seem to be more susceptible to experiencing stress caused by the combination of high demands and poor relationships, which may be the symptoms of workplace discrimination or bullying [44, 45]. High demands noted in this group may be related to the potential unfair treatment of unmarried workers, such as giving them extra tasks and expecting them to be more available and ready to take night shifts, work overtime and on holidays than married workers [45]. Interestingly, in a study by Kazi and Haslam [24] on a sample of call center employees, Demands and Relationships were the only subscales of the HSE-MS IT negatively related to psychological well-being measured with the General Health Questionnaire-12 (GHQ-12). In light of the above, although Profile 5 was small (5.2%), further research on its specificity and mental functioning is highly recommended.

## Practical implications

Considering the high prevalence of stress, anxiety, fatigue, and depression in healthcare workers caring for COVID-19 patients [6, 8, 12] and the fact that healthy and motivated healthcare workers are vital for any health system, monitoring work-related stress in this group is highly needed. The HSE-MS IT seems to be a suitable, convenient, and psychometrically sound measure for this purpose, providing a broad overview of sources of work-related stress within organizations that may help quickly assess occupational stress in critical situations and in taking appropriate actions and mitigating complications of prolonged stress.

Using LPA may help recognize different stress profiles among healthcare workers, which may provide important cues for focused interventions and stress reduction programs better tailored to the needs of workers than “one size fits all” solutions [23, 46]. For example, in the current study, the LPA led to identifying the profile that grouped people who are relatively highly stressed at work despite average levels of demands at the workplace (Profile 3). This result suggests that stress may not only occur when there is a mismatch between job demands and resources but may also result from poor organizational and/or personal resources, such as the lack of control, support, the ambiguity of job role, or poorly managed changes in the organization. Following this, interventions aimed at improving personal and work-related resources should not only be treated as the counterbalance for high job demands but as valuable *per se* since their lack or insufficiency may lead to high perceived stress.

Moreover, when preparing interventions for healthcare workers, it can be useful to pay attention to sociodemographic and work-related predictors of profile membership found in this study. For example, the uniform Profile 4 (“low stress”) grouped younger employees than the more diversified Profiles 1 (“high stress with a good understanding of one’s job role”) and 5 (“generally low stress but with very high job demands and relational conflicts”); similarly, the uniform Profile 2 (“moderate stress”) consisted of younger employees than the more diversified Profile 1. This suggests that older healthcare workers may have a greater tendency for work-related stressors differentiation than younger ones and may feel stressed due to some of them but not to others, whereas the younger ones perceive work-related stressors more uniformly. It may mean that age-related differences should be considered when developing stress management programs for healthcare workers. Another interesting result concerning the predictors of profile membership is that the proportion of ED nurses to EMS staff was higher in Profile 3 (“relatively high stress with average demands and a very low understanding of one’s job role”) than in Profile 1 (“high stress with a good understanding of one’s job role”). This result suggests that during the pandemic, ED nurses may be more prone to having unclear, ill-defined job roles and have to carry out tasks beyond their regular duties more often than EMS staff; thus, the clarification of job responsibilities and role transparency seems to be especially needed in this group [22].

## Limitations

This study has several considerable strengths: identifying various constellations of work-related stress and their sociodemographic and work-related predictors among healthcare workers, collecting a relatively large sample with an adequate response rate [47], using a standard, widely known questionnaire to assess work-related stress, and making comparisons between ED nurses and EMS staff. Despite these strengths, the present study also has some limitations that should be discussed.

First, due to the cross-sectional nature of the data, we cannot draw definite conclusions about the directionality of associations between the identified profiles and covariates. However, the examined predictors of profile membership are well-grounded in the work-related stress studies that applied the variable-centered approach. Second, due to the COVID-19 restrictions and related difficulties with reaching EDs and EMS centers across the country, the study was conducted on a convenience sample of ED nurses and EMS staff in one province in Iran, which limits its generalizability. Third, no outcomes of latent profile membership were considered in the present study. Future studies may examine potential consequences of profile membership (such as well-being, burnout, job satisfaction, or life satisfaction). Fourth, despite certain advantages of using HSE-MS IT to measure work-related stress, it is a self-report, subjective measure in which participants’ responses may be affected by their mental states. Future studies may include multiple informants’ reports to measure healthcare workers’ levels of work-related stress.

## Conclusion

The results of this study show that during the COVID-19 pandemic, the sample of Iranian healthcare workers could be grouped into five qualitatively distinct profiles in terms of work-related stress, which were either uniform or diversified depending on the stress-related domain. Age, marital status, service location, workplace, and overtime predicted profile membership. The study points to the need to assess multiple aspects of work-related stress of healthcare workers and the benefits of implementing LPA, which gives a complex picture of occupational stressors and thus may help prepare tailored interventions to reduce the level of work-related stress in this group.

## Data Availability

The data that support the findings of this study are available from the corresponding author [A.M.] upon request.
